# Inspection of *Capparis spinosa* essential oils for quality assurance of fish burgers during refrigerated storage

**DOI:** 10.1002/fsn3.3648

**Published:** 2023-09-07

**Authors:** Mohammad Shafaghi Rad, Marjan Nouri

**Affiliations:** ^1^ Department of Food Science and Technology, Roudehen Branch Islamic Azad University Roudehen Iran

**Keywords:** antioxidant, burger, *Capparis spinosa*, extraction method, microbial spoilage

## Abstract

Fish products are highly perishable as a result of easy spoilage by microorganism populations. The aim of this study is to evaluate the effects of *Capparis spinosa* essential oils (CSEOs) on physicochemical, sensory, oxidative, and microbiological attributes for fish burgers during refrigerated storage (8 days). For this purpose, CSEOs were prepared by water distillation (CS‐WD), CO_2_ supercritical fluid (CS‐SCF), subcritical water (CS‐SW) to determine bioactive substances. Total phenol and flavonoid contents and also antioxidant activity were measured in CSEOs extracted by these procedures and phytochemical identification was performed through gas chromatography and mass spectroscopy. These essential oils (EOs) were used at 0.2% in fish burgers, and controls (without additives) and those containing sodium erythorbate were also prepared. Physicochemical, oxidative, microbiological, and sensory functions were examined every 2 days. Phytochemicals were found in CSEOs, and the highest was related to isopropyl isothiocyanate. Addition of CSEOs led to dark, yellow and red colors for fish burgers compared with control. The manufacture of primary and secondary products in oxidation and different microorganisms in samples were significantly decreased by CSEOs compared with controls (*p* < .05). The antioxidant feature of the burger with CS‐SCF was higher than that of the sample treated with CS‐WD, but these EOs indicated almost the same antimicrobial attributes, and the lowest antioxidant and microbial activities were found for CS‐SW in the fish burger. The sensory results demonstrated that CSEOs did not reduce scores, which maintained the acceptance quality of burgers during refrigerated storage. As a conclusion, CSEOs can be used as effective antioxidants and preservatives in burgers, and sensory acceptance was preserved during storage.

## Graphical abstract

Capparis spinosa essential oils can be used as effective antioxidants and preservatives in burgers, and sensory acceptance was preserved during storage.

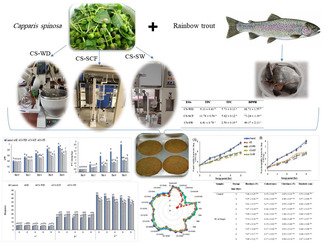

## 1 INTRODUCTION

Fish is a valuable food that includes omega‐3 and omega‐6 unsaturated fatty acids, proteins with high biological values, and mineral salts (El‐Ghareeb et al., 2021). These functional components are essential and contribute to human health, especially for the heart and brain (Delfino et al., 2023). It is necessary to develop different ready‐to‐eat products due to the noticeable role of fish in human nutrition in order to enhance consumption by creating a higher level of diversity (Abbey et al., 2017; Rico et al., 2020). Fish and its products are highly perishable foods owing to the easy growth of spoilage microorganisms and their limited shelf life (El‐Ghareeb et al., 2021; Ji et al., 2021). Processes, handling, and storage can improve the safety and quality of fishery products under appropriate conditions (Abbey et al., 2017; Dolea et al., 2018). However, it is necessary to use additional technologies or preservative additives to extend the shelf life of these perishable products (Shahbazi & Shavisi, 2018).

Preservatives are chemical additives that can improve stability, quality, and shelf life, as well as reduce the microbial load and economic losses of edible products (Delfino et al., 2023; Mooliani & Nouri, 2021). Nevertheless, adverse effects of these chemical preservatives, such as allergenicity and carcinogenicity, have been confirmed on human health (Delfino et al., 2023; Hashemi et al., 2022). Therefore, natural alternatives are substituted for chemical and synthetic additives in the food industry because of consumer awareness (Farnejad et al., 2022). Plant essential oils (EOs) are considered among the most discussed natural additives to extend shelf life and maintain the quality of food products (Nouri, 2022). In previous studies, EOs and extracts from different plant sources had been successfully used to preserve the oxidative and microbiological quality of fish and its products (Delfino et al., 2023; Hashemi et al., 2022; Jooyandeh & Yadmellat, 2018; Maghami et al., 2019; Pourmollaei et al., 2021).

*Capparis spinosa* (CS) is a perennial and medicinal plant in the Mediterranean region that belongs to the *Capparaceae* family and also grows in North Africa, Central Asia, Italy, Greece, and Iran (Kalantari et al., 2018). The different parts of CS are applied in traditional medicine, and its fruit has a green outer layer in all stages of plant growth (Allaith, 2016; Jiménez‐López et al., 2018). This fruit contains flavonols, flavonoids, phenols, tannins, steroids, triterpenoids, and saponins, so research has shown that CS is a rich source of sulfurs and glycosides (Hematian et al., 2020). The prominent medicinal and biological applications of CS include anticancer, antioxidant, antimicrobial, cardiovascular, hepatoprotective, and hypoglycemic attributes (Jiménez‐López et al., 2018).

Bioactive components of plants have different chemical structures and polarities; therefore, various approaches have been developed to extract EOs from plant sources (Delfino et al., 2023). Some of the extraction methods include ultrasonic (Fard & Nouri, 2023), enzymatic (Fan et al., 2019), microwave (Jafari et al., 2019), CO_2_ supercritical fluid (CS‐SCF; Abdolahi et al., 2023), high pressure (Alonso‐Riaño et al., 2020), subcritical water (CS‐SW; Salami et al., 2020), etc. So, in the present study, the effect of distinct extraction procedures on the bioactive components and antioxidant traits of *Capparis spinosa* essential oils (CSEOs) were investigated. Then, the application of CSEOs was examined to improve the quality and shelf life of rainbow trout fish burgers during refrigerated storage (4°C).

## 2 MATERIALS AND METHODS

### 2.1 Materials

CS fruit was prepared from the local market in Tehran (Iran), which was powdered after drying in an oven under vacuum conditions at 40°C. Rainbow trouts with an approximate weight (650 ± 100 g) and length (5 ± 1 cm) were purchased from a fish breeding center and transported to the laboratory using ice buckets. The formulation ingredients for fish burgers were obtained from the local supermarket, as were the chemicals and culture medium from Merck Company of Germany.

### 2.2 Preparation of CSEOs

The CS fruit powder (100 g) was poured into a 2 L balloon connected to the Clevenger apparatus, and distilled water (1000 mL) was added to extract CSEO by the water distillation (WD) method. The extraction process continued for 4 h when water boiled inside the balloon, and CSEO was collected in small jars and dehydrated by anhydrous sodium sulfate (Rico et al., 2020).

CS fruit powder (20 g) was mixed with ethanol solvent (100 mL), and the extraction procedure was performed for 30 min at a pressure of 100 bar and 35°C to extract CSEO by CS‐SCF with a flow rate of 1 mL/min (Suprex MPS/225 Multipurpose system). After this process, the solution was centrifuged at 300 rpm for 10 min, and the supernatant was collected at each stage so that no more sediment was seen at the tube bottom. Then, the supernatant was collected and filtered with Whatman filter paper NO. 1, and the solvent was vaporized by the evaporator under vacuum at 50°C. CSEO was extracted by CS‐SW when CS fruit powder (12 g) was transferred into the device containing glass beads (Abdolahi et al., 2023).

#### 2.2.1 Determination of total phenol content (TPC), total flavonoid content (TFC), and antioxidant activity of CSEOs

Briefly, the sample (0.1 mL), distilled water (2.8 mL), and Folin–Ciocalteau reagent (0.1 mL) were mixed together and vortexed to determine the TPC of CSEOs. Afterwards, 7.5% (w/w) sodium carbonate solution (2 mL) was stirred with the mixture, then incubated for 60 min in the dark (25°C) and finally absorbance was recorded using a spectrophotometer at 750 nm (Jenway 6305, England). The standard curve of gallic acid (GA) was plotted, and TPC was reported as milligrams of GA equivalents per gram (mg GAE/g) for samples (Alonso‐Riaño et al., 2020).

A sample (0.5 mL) was mixed with absolute ethanol (1.5 mL), 0.1 M CH_3_COOK solution (0.1 mL), 10% (w/v) AlCl_3_ solution (0.1 mL), and distilled water (2.8 mL), which was kept for 30 min to determine the TFC of CSEOs. After that, the mixture was filtered, and the absorbance was read at 415 nm using a spectrophotometer. A standard curve of quercetin in ethanol was plotted, and TFC was calculated as milligrams of quercetin equivalent (QE) per gram (mg QE/g) of CSEOs (Aminzare et al., 2022).

A 0.3 mL of sample was vigorously mixed with 2.7 mL of methanolic solution (0.004%) to measure the antioxidant activity of CSEOs by the 1‐(2, 6‐dimethylphenoxy)‐2‐(3, 4‐dimethoxyphenylethylamino (DPPH) free radical scavenging method. The light absorbance was read at 517 nm against a blank (without sample) after 60 min of incubation at room temperature and darkness, and the DPPH radical scavenging percentage was calculated using Equation (1) (Hashemian & Nouri, 2023):

(1) $$\text{DPPH radical scavenging}\;\left(\%\right)=\frac{A\left(\text{blank}\right)-A\;\left(\text{sample}\right)}{A\left(\text{blank}\right)}\times 100$$

#### 2.2.2 Phytochemical identification of CSEOs by gas chromatography and mass spectroscopy (GC–MS)

The GC–MS method (TRACE MS, America) with a capillary column (30 m × 0.25 mm × 0.25 μm) was applied to assess the phytochemicals of CSEOs. The thermal program was considered from 50 to 270°C with an enhancement of about 3°C per min, and the temperature of the injection chamber and oven was set to 250°C. Helium gas was used as a carrier gas with a high purity and flow rate of 1 mL/min, as well as an ionization energy and injection volume of 70 eV and 0.2 μL, respectively. The phytochemical constituents for CS was identified by retention indexes and checking mass spectra was available in computer libraries and valid references compared to standard data (Noshad et al., 2021).

### 2.3 Preparation of fish burgers

The fish burger formulations consisted of 85% minced fillet, 5% breadcrumb powder, 0.5% sugar, 2% salt, 4% onion powder, 1.5% garlic powder, and 2% spices, including turmeric, pepper, and curry. After separating the head, bones, and skin, the fish was washed with water and also cut into fillets, which were grounded three times by a meat grinder and formulation ingredients were added until a homogeneous batter was produced. CSEOs and sodium erythorbate (SE) as additives are added to the batter at a 0.2% level; the samples are illustrated in Table 1. The prepared batters were shaped by manual molding into burgers with a weight of approximately 100 ± 5 g and 1 cm thickness. The burgers were packed in polyethylene bags and kept at 4°C, and the tests were performed on samples every two days (Hashemi et al., 2022).

**TABLE 1:** Abbreviations of different methods for essential oil extraction, along with pictures of produced burgers.

Name	Essential oils	Pictures of treatments
Control	No additive	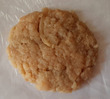
SE	Sodium erythorbate	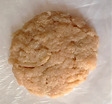
CS‐WD	*Capparis spinose* essential oil obtained by water distillation	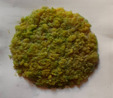
CS‐SCF	*Capparis spinose* essential oil obtained by CO_2_ supercritical fluid	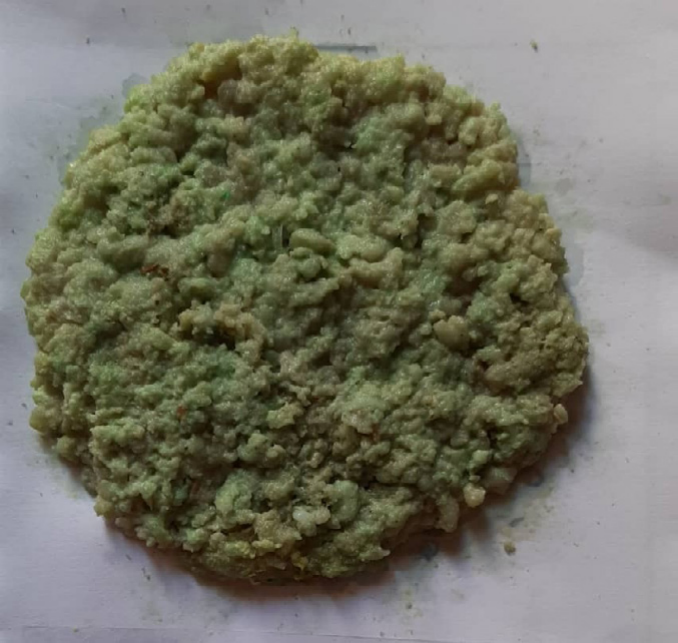
CS‐SW	*Capparis spinose* essential oil obtained by subcritical water	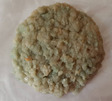

#### 2.3.1 Physicochemical assessments

To measure burger pH, 5 g of each sample was mixed with 45 mL of distilled water for 60 s, then filtered, and, finally, the obtained result was measured by a pH meter (Zenit, Germany) at 23 ± 2°C.

Total volatile base nitrogen (TVB‐N, mg N 100 g^−1^) was measured, and 10 g of fish burgers were added to a Kjeldahl flask containing magnesium oxide, and then mixed with distilled water before connecting to the system. The fluid was placed and collected in an Erlenmayer flask, including boric acid (2%) and methyl red indicator through 10 N H_2_SO_4_ that changes color from green to light red; after that, TVB‐N was determined in a 100 g fish burger as follows (Mahmoudzadeh et al., 2010):

$$\begin{array}{cc} \mathrm{TVB}-N\;\left(\mathrm{mg}\;N\;100\;g^{-1}\right)\; & =1.4\times \text{used}\;H_{2}{\mathrm{SO}}_{4}\times 100\\ & \times \text{amount of sample}/1000\;\mathrm{mg} \end{array}$$

#### 2.3.2 Oxidative index measurement

Initially, 40 g of fish burger was mixed with 100 mL of chloroform and filtered by Whatman filter paper No. 44 for determining peroxide value (PV). Then, 25 mL of filtered solution was separated by a rotary evaporator (Heidolph, Germany), and 5 g extracted fat was poured into a 250 mL Erlenmeyer flask. Then, 37 mL of acetic acid–chloroform solution (2:3 ratio), 1 mL of saturated potassium iodide, and 30 mL of distilled water with 1 mL of starch solution after 60 s were added. The released iodine was titrated with sodium thiosulfate solution (0.01 N) until yellow disappeared and a milky color was created; also PV was also reported as milliequivalents per kilogram (meq/kg) for samples (Amiri et al., 2019).

Thiobarbituric acid (TBA) was measured for burgers when 1 g of each sample was transferred to a Falcon tube, and after that, 3 mL of perchloric acid (4%) and 2 mL of trichloroacetic acid (10%) were added. After closing the lid of the tubes, they were stirred for 3 min and then centrifuged at 4000 rpm for 15 min to settle the suspended substances in the extracts. Afterwards, 1 mL of supernatant was transferred to a test tube, and 1 mL of TBA reagent (0.02 M) was added. After heating for 50 min at 90°C with the appearance of a pinkish‐orange color, a cooled solution was obtained, and absorbance was read at 530 nm against a blank (including all test materials except the sample). The TBA for burgers was reported as milligrams of malondialdehyde (MDA) per kilogram (mg MDA/kg) (Maghami et al., 2019).

#### 2.3.3 Color analysis

The color parameters, including L* (brightness), a* (redness‐greenness), and b* (yellowness‐blueness), were determined using a Hanterlab colorimeter (Minolta, Japan) for fish burgers (Rico et al., 2020).

#### 2.3.4 Textural profile analysis (TPA)

The TPA of fish burgers was done using a texture analyzer (LLOYD, RS 232, America) equipped with a 250 N cell load and a cylindrical probe with a flat end (7.5 cm diameter). The cube‐shaped slices (2 cm × 2 cm) were prepared from the center of burgers, and compression speed was calculated at 1 mm/s by the probe of device. Also, the samples were compressed to 50% of their initial height. In the present research, the texture parameters were investigated such as hardness (N), cohesiveness, chewiness (N), and elasticity (cm) (Dolea et al., 2018).

#### 2.3.5 Microbiological analysis

Initially, a 10 g burger sample was aseptically homogenized in 90 mL of 0.1% sterile peptone solution for 120 s at maximum speed in a Stomacher. The prepared diluted samples were serially diluted at a ratio of 1:10 in peptone; after that, a 100 μL sample dilution was surface‐plated onto culture media and incubated. The culture media and incubation conditions for each microorganism were as follows: total viable bacteria count: plate count agar (PCA) medium, incubation temperature (30°C), and time (48 h); psychrophilic bacteria: PCA media, 7°C for incubation temperature, and time about 10 days; coliforms: red bile violet agar (RBVA) media, incubation temperature, and time of 37°C for 24 h; lactic acid bacteria (LAB): De man, Rogosa, and Sharpe agar (MRS) media, 37°C for incubation temperature, and about 24 h (Emiroğlu et al., 2010); and molds and yeasts: potato dextrose agar (PDA) media and incubation temperature about 25°C during 120 h (Emiroğlu et al., 2010; Olivas‐Méndez et al., 2022).

#### 2.3.6 Sensory evaluation

The sensory evaluation of fish burgers was done by 30 panelists (university students; 15 men and 15 women) according to a 5‐point hedonic test (5 = very good and 1 = very bad). The sensory characteristics investigated were texture, flavor, color, odor, and overall acceptability. The burgers were cooked at 150°C on a hot plate, and the core temperature of samples was 72°C (Çoban & Keleştemur, 2017).

### 2.4 Statistical analysis of data

In the present research, tests were done in triplicates, and results were statistically analyzed using the statistics software of SPSS 22.0. The ANOVA (one‐way) and Duncan's multiplication test at *p* < .05 (a statistical significance of 95%) were used to assess the data.

## 3 RESULTS AND DISCUSSION

### 3.1 TPC, TFC, antioxidant activity, and chemical composition of CSEOs

The results of TPC, TFC, and antioxidant activity for CSEOs extracted by different methods are presented in Table 2. The highest TPC and DPPH radical scavenging were observed for the obtained sample with CS‐SCF (11.78 mg GAE/g and 73.24%), followed by WD (9.13 mg GAE/g and 68.71%), and the lowest values were obtained in the treatment achieved by CS‐SW (6.41 mg GAE/g and 49.37%), respectively. The highest TFC was detected in fish burgers treated with CS‐SCF (5.62 mg QE/g) and WD (5.73 mg QE/g), so there was no significant difference between these samples. The lowest TFC was also observed in fish burgers prepared with CS‐SW (2.50 mg QE/g), which was similar to TPC.

**TABLE 2:** Total phenol (TPC**)**, flavonoid contents (TFC**)**, and antioxidant activity (DPPH radical scavenging**)** of CSEOs.

Essential oils	TPC (mg GAE/g)	TFC (mg QE/g)	DPPH radical scavenging (%)
CS‐WD	9.13 ± 0.41^b^	5.73 ± 0.13^a^	68.71 ± 1.55^b^
CS‐SCF	11.78 ± 0.56^a^	5.62 ± 0.12^a^	73.24 ± 1.39^a^
CS‐SW	6.41 ± 0.78^c^	2.50 ± 0.19^b^	49.37 ± 2.13^c^

*Note*: Values represent the mean (*n* = 3) ± *SD*. Different letters in each column represent significant differences at 5% level of probability among samples.

Abbreviations: CS‐SCF, *Capparis spinose* essential oil obtained by CO_2_ supercritical fluid; CS‐SW, *Capparis spinose* essential oil obtained by subcritical water; CS‐WD, *Capparis spinose* essential oil obtained by water distillation.

In the previous studies, distinct values of TPC, TFC, and antioxidant function were reported for CS fruit; for instance, TPC and TFC of CS grown in Bahrain were expressed as 120.08 mg GAE/100 g and 39.96 mg RE/100 g, respectively (Allaith, 2016). TPC (555.0 mg GAE/g) and TFC (102.6 mg RE/g) were calculated for the methanolic extract of CS (Essien et al., 2020). The levels of TPC (6.5 mg GAE/g extract), TFC (2.42 mg QE/g extract), and DPPH radical scavenging (0.98 g TE/100 g extract) in CS were determined (Jiménez‐López et al., 2018). *Lavandula* extract had a higher total phenolic content (9.8 ± 0.2 mg GA.g^−1^ of extract) and antioxidant activity (34 ± 7 g of extract.g^−1^ of DPPH) than *Moringa* leaf extract (2.2 ± 0.1 mg GA.g^−1^ of extract) and (689 ± 22 g of extract.g^−1^ of DPPH), respectively (Delfino et al., 2023). The results of the present study also illustrated that the extraction approach had a significant impact on TPC, TFC, and the antioxidant potential of CS fruit (*p* < .05). Although the CS‐SW method is a fast extraction process due to the use of high temperatures, heat‐sensitive bioactive compounds can be destroyed, and this method is more effective for extracting polar substances (Salami et al., 2020). However, samples containing CS‐SCF caused the least damage to bioactive materials and non‐polar components, and some with medium polarity were easily extracted (Essien et al., 2020). Thus, the CS‐SCF method often works better for extracting active constituents from plant sources (Abdolahi et al., 2023). The lower TPC of pumpkin peel extract was obtained for samples using CS‐SW compared with CS‐SCF (Salami et al., 2020), which is in line with present research.

The phytochemicals of prepared CSEOs from different methods are presented in Table 3, of which 12 chemical substances were found in CSEOs. In samples of CS‐WD, CS‐SCF, and CS‐SW, the highest level was related to isopropyl isothiocyanate (66.70%–77.92%), and other major phytochemicals were found in CSEOs, including isobutyl isothiocyanate (4.61%–6.14%), butyl isothiocyanate (4.88%–5.11%), and thymol (2.34%–9.39%). The amount of γ‐terpinene (3.39%), p‐cymene (2.25%), camphor (5.87%), and carvone (4.12%) was also remarkable for the sample in CS‐SW. Similarly, isopropyl isothiocyanate was identified as the main composition of CSEO (Hematian et al., 2020). The isopropyl isothiocyanate, methyl sulfonyl heptyl isothiocyanate, butyl isothiocyanate, γ‐terpinene, and thymol were detected as major constituents of Iranian CS fruit (Allaith, 2016; Jiménez‐López et al., 2018).

**TABLE 3:** Phytochemical identification for CSEOs by GC–MS.

RI	Compounds	Values in CS‐WD (%)	Values in CS‐SCF (%)	Values in CS‐SW (%)
877	Ethyl thiocyanate	0.82	0.69	0.70
885	Isopropyl isothiocyanate	68.57	77.92	66.70
946	Butyl isothiocyanate	5.07	5.11	4.88
966	Isobutyl isothiocyanate	6.14	5.60	4.61
1031	p‐Cymene	0.04	0.07	2.25
1033	Limonene	0.05	0.05	0.48
1037	1,8‐Cineole	0.15	0.03	0.02
1062	γ‐Terpinene	0.24	0.05	3.39
1108	Para‐menta‐1(7),8‐dien	0.39	0.08	0.01
1158	Camphor	0.46	0.06	5.87
1316	Carvone	0.73	0.95	4.12
1352	Thymol	2.34	9.39	6.97

Abbreviations: CS‐SCF, *Capparis spinose* essential oil obtained by CO_2_ supercritical fluid; CS‐SW, *Capparis spinose* essential oil obtained by subcritical water; CS‐WD, *Capparis spinose* essential oil obtained by water distillation.

### 3.2 The pH of burgers

**FIGURE 1:**
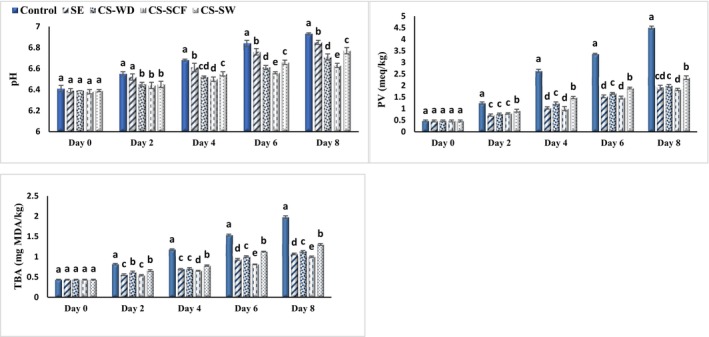
Changes in the pH, PV, and TBA values of fish burgers containing different essential oils during the storage period. Bars represent the mean (*n* = 3) ± *SD*. Different letters on the bars indicate significant differences at the 5% level of probability among samples at the same time. CS‐SCF, *Capparis spinose* essential oil obtained by CO_2_ supercritical fluid; CS‐SW, *Capparis spinose* essential oil obtained by subcritical water; CS‐WD, *Capparis spinose* essential oil obtained by water distillation; SE, sodium erythorbate.

The results of pH for fish burgers outlined that a significant effect was not indicated by samples containing 0.2% SE and CSEOs on this feature at the beginning of storage, which were in the range of 6.38–6.41 (Figure 1). The pH values of samples increased significantly over time (*p* < .05), which was related to the decomposition of proteins by internal protease and the enzymes secreted through the bacteria (especially psychrophilic). As a result of protein decomposition, volatile nitrogen substances such as ammonia and trimethylamine are formed, which enhance the pH of the product (Tanavar et al., 2021).

Protease activity was enhanced by a higher microbial load; as a result, control had the highest pH; however, this feature was elevated at a significantly slower rate (*p* < .05) in treated samples by SE, CS‐WD, CS‐SCF, and CS‐SW due to lower bacteria growth and less breakdown of proteins. The highest pH value on the last day of storage was for control (6.93), and the lowest was obtained for used CS‐SCF in burger (6.63). The pH of fish burgers was elevated by storage due to alkaline formations, and in others, it declined owing to the degradation of fish muscles (Delfino et al., 2023), and an increase in this level was found for catfish burgers during the shelf life (Salami et al., 2020). The addition of fennel EOs to fish burgers led to a reduction in the intensity of alkaline production, and as a result, pH was enhanced at a lower rate in treated burgers compared to control (Rico et al., 2020), which was in agreement with the present results.

### 3.3 The TVB‐N levels of burgers

At the beginning of shelf life, TVB‐N levels were measured as 13.41 (mg N 100 g^−1^) for control and near 13.39 (mg N 100 g^−1^) for treated burgers using SE and CSEOs. These levels were elevated considerably 23.93 (mg N 100 g^−1^) for control at the end of shelf life; however, they increased slower in treated burgers after the 8th day. The significantly lower levels (*p* < .05) of TVB‐N were achieved in treated samples compared to controls during shelf life; but, no significant differences (*p* > .05) were found among all. The TVB‐N did not exceed the acceptability limit of 35 (mg N 100 g^−1^) for each groups; the quality of fish and its products would be ‘high’, with values up to 25 (mg N 100 g^−1^) for all present samples in this range (Uçak et al., 2011). While, lower TVB‐N were obtined in treated burgers with rosemary extract (Uçak et al., 2011), *Mentha spicata* EO and zinc oxide nanoparticle (Shahbazi & Shavisi, 2018) and water lily extracts (Dulal et al., 2023). This is corresponded to effect of applied extract on bacteria population as antimicrobial agent (Uçak et al., 2011).

### 3.4 The PV index of burgers

Hydroperoxides are odorless substances and primary oxidation products of fats and oils, and peroxide is an index that represents primary products resulting from oxidation (Noshad et al., 2021; Salami et al., 2020). In general, PV was considered as a desirable factor when its level was less than 2 meq/kg for foods, which should not exceed 5 meq/kg (Guran et al., 2015). The results of PV evaluation for fish burgers are given in Figure 1; at the beginning of storage, PV was 0.47 meq/kg. The PV of all burger samples increased significantly with the passage of time due to fat oxidation and hydroproxide production; the highest PV was observed in control since it had no preservative additive (*p* < .05). CSEOs were able to remarkably reduce the rate of hydroproxides production, and on account of the higher phenolic contents of CS‐SCF in fish burgers, treated samples indicated higher antioxidant ability than others (Figure 1). At the end of storage, control had the most PV (4.50 meq/kg) and the lowest level was for samples treated using CS‐SCF (1.83 meq/kg); nevertheless, no significant difference was observed between this sample and burgers containing SE. The SEOs indicated protection against primary and secondary manufactures for lipid oxidation in fresh rainbow trout fillets during their 7 days of shelf life. Polyphenols are actually able to inhibit free radicals, especially peroxy radicals, which are among the most reactive reactants in the chain of fat oxidation, and terminate this cycle, resulting in fewer hydroperoxides being formed (Fard & Nouri, 2023; Jiménez‐López et al., 2018). The antioxidant capability of different parts of CS has been confirmed in previous research (Allaith, 2016; Hematian et al., 2020; Kalantari et al., 2018). A significant decrease was reported in hydroperoxide formations for fish burgers containing chitosan nanoparticles and fennel EOs (Maghami et al., 2019). Similarly, the noticeable effect of plant extracts of thyme, sage, and green tea on PV reduction was observed in fish burgers (Ozogul & Uçar, 2013).

### 3.5 The TBA index of burgers

TBA measures the formation of secondary products for fat oxidation, especially MDAs (Rico et al., 2020). These components are caused by the breakdown of hydroperoxides formed in the first stage of fat oxidation, which create a color complex due to reaction with TBA reagent (Ozogul & Uçar, 2013; Tanavar et al., 2021). Since fish and its products contain high amounts of polyunsaturated fatty acids (PUFAs), their sensitivities to oxidative spoilage are high, and the maximum TBA for these products is 5 mg MDA/kg (Guran et al., 2015; Mattje et al., 2019). The changes in TBA of fish burgers during the refrigerated period are outlined in Figure 1, and these values for samples were close to 0.435 mg MDA/kg at the beginning of storage. TBA was enhanced considerably over time for samples owing to the progress of fat oxidation and higher secondary products (*p* < .05). As expected, the burger without additive (control) had the highest TBA (1.976 mg MDA/kg), and the lowest was observed in the sample treated with CS‐SCF (1.000 mg MDA/kg) on the last day of storage. The phenolics in plant sources have hydroxyl groups in their structures, which inhibit free radicals and thereby prevent the fat oxidation process or reduce the speed of this destructive reaction (Aminzare et al., 2022; Salami et al., 2020). Overall, phenolic compositions are the main agents in inhibiting free radicals; however, other bioactive substances such as polysaccharides, carotenoids, proteins, peptides, and pigments in some plants can also be involved in neutralizing free radicals (Noshad et al., 2021). In agreement with the present results, the antioxidant effect of ginger subcritical extract and EOs was also reported in delaying oxidative spoilage of tilapia fish burgers (Mattje et al., 2019). The effect of water lily extracts on oxidative stability indicated higher TBA in treated tilapia fish fillets compared with control on account of phenolic presence (Dolea et al., 2018).

### 3.6 The color of burgers

**FIGURE 2:**
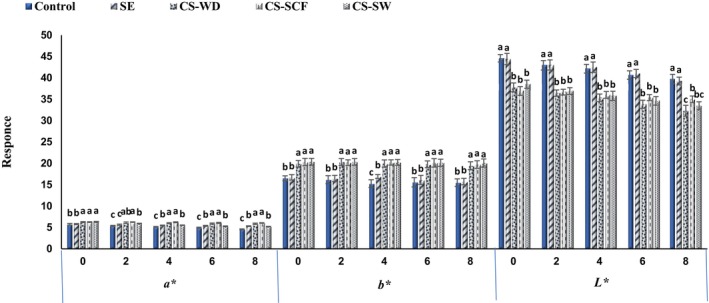
Changes in color indexes of fish burgers containing different essential oils during the storage period. Bars represent the mean (*n* = 3) ± *SD*. Different letters on the bars indicate significant differences at the 5% level of probability among samples at the same time. CS‐SCF, *Capparis spinose* essential oil obtained by CO_2_ supercritical fluid; CS‐SW, *Capparis spinose* essential oil obtained by subcritical water; CS‐WD, *Capparis spinose* essential oil obtained by water distillation; SE, sodium erythorbate.

The color is one of the most obvious quality indicators for product marketability, and this parameter in fish burgers, including L*, a*, and b*, is determined by the calorimetric method, whose results are illustrated in Figure 2. On all the days studied in the present research, the burgers containing CSEOs had less brightness with more redness and yellowness than others (*p* < .05). The redness of burgers gradually decreased (*p* < .05), while CSEOs could reduce the severity of redness for treated burgers compared with control during refrigerated storage. The brightness of the color gradually decreased with time, but no significant change was distinguished in the yellowness of the samples. L*, a*, and b* were in the range of 36.93–44.58, 5.74–6.30, and 16.52–20.37 for burgers at the beginning of storage, which reached 32.30–39.74, 4.55–6.04, and 15.48–20.08 on the last day, respectively.

A decrease in brightness for fish burgers was reported over time, and the addition of fennel extract to formulations reduced lightness and also increased redness and yellowness (Rico et al., 2020). The discolored meat and its products were observed because of fat oxidation and also primary and secondary components of synergistic phenomena, which are created by passage of time and also react with iron ion in structure of oxymyoglubin and metmyoglubin pigments (Dolea et al., 2018; Jooyandeh & Yadmellat, 2018). The reduction in L* and a* enhancement was also reported in salmon fish burgers due to the addition of oregano and clove EOs (Jonušaite et al., 2021). The fish burger with *Moringa* and *Lavandula* extracts illustrated higher colors owing to intensity (Delfino et al., 2023).

### 3.7 TPA of burgers

The TPA such as hardness, cohesiveness, chewiness and elasticity of fish burgers were elevated by a texture analyzer, which the results are displayed in Table 4. No considerable change in the textural parameters of burgers during refrigerated storage, and only a slight increase in hardness and chewiness, was observed, which was not statistically significant. The hardness, cohesiveness, chewiness, and elasticity for fish burgers were in the range of 5.40–5.89 N, 0.35–0.39, 0.84–1.06 N, and 0.50–0.68 cm, respectively. In agreement with these results, a significant effect was not detected on textural parameters by adding thyme and oregano EOs to the formulation of salmon fish burger (Dolea et al., 2018). The addition of *Mentha spicata* EO and zinc oxide nanoparticles and also over time had no effect on the texture of rainbow trout fillets (Shahbazi & Shavisi, 2018).

**TABLE 4:** Comparison of the texture profile (hardness, cohesiveness, chewiness, and elasticity) of fish burgers during the storage period.

Samples	Storage time (day)	Hardness (N)	Cohesiveness	Chewiness (N)	Elasticity (cm)
Control	0	5.40 ± 0.25^A, a^	0.35 ± 0.02^A, a^	0.85 ± 0.10^A, a^	0.53 ± 0.08^A, a^
	2	5.47 ± 0.22^A, a^	0.36 ± 0.05^A, a^	0.90 ± 0.07^A, a^	0.57 ± 0.09^A, a^
	4	5.61 ± 0.15^A, a^	0.37 ± 0.03^A, a^	0.95 ± 0.09^A, a^	0.62 ± 0.06^A, a^
	6	5.67 ± 0.14^A, a^	0.36 ± 0.02^A, a^	0.97 ± 0.09^A, a^	0.67 ± 0.10^A, a^
	8	5.89 ± 0.24^A, a^	0.36 ± 0.03^A, a^	1.06 ± 0.12^A, a^	0.68 ± 0.08^A, a^
SE in burger	0	5.53 ± 0.18^A, a^	0.36 ± 0.03^A, a^	0.86 ± 0.09^A, a^	0.57 ± 0.07^A, a^
	2	5.45 ± 0.16^A, a^	0.35 ± 0.02^A, a^	0.89 ± 0.06^A, a^	0.60 ± 0.06^A, a^
	4	5.47 ± 0.20^A, a^	0.37 ± 0.02^A, a^	0.93 ± 0.12^A, a^	0.64 ± 0.08^A, a^
	6	5.67 ± 0.17^A, a^	0.37 ± 0.03^A, a^	0.93 ± 0.07^A, a^	0.64 ± 0.05^A, a^
	8	5.60 ± 0.21^A, a^	0.37 ± 0.02^A, a^	0.96 ± 0.14^A, a^	0.67 ± 0.09^A, a^
CS‐WD in burger	0	5.47 ± 0.12^A, a^	0.36 ± 0.01^A, a^	0.84 ± 0.12^A, a^	0.50 ± 0.10^A, a^
	2	5.40 ± 0.19^A, a^	0.37 ± 0.02^A, a^	0.86 ± 0.09^A, a^	0.53 ± 0.11^A, a^
	4	5.41 ± 0.22^A, a^	0.39 ± 0.04^A, a^	0.91 ± 0.11^A, a^	0.56 ± 0.07^A, a^
	6	5.58 ± 0.23^A, a^	0.39 ± 0.02^A, a^	0.94 ± 0.08^A, a^	0.59 ± 0.08^A, a^
	8	5.58 ± 0.28^A, a^	0.38 ± 0.03^A, a^	0.93 ± 0.10^A, a^	0.63 ± 0.12^A, a^
CS‐SCF in burger	0	5.48 ± 0.20^A, a^	0.35 ± 0.04^A, a^	0.86 ± 0.14^A, a^	0.55 ± 0.09^A, a^
	2	5.57 ± 0.17^A, a^	0.36 ± 0.03^A, a^	0.89 ± 0.07^A, a^	0.57 ± 0.07^A, a^
	4	5.53 ± 0.19^A, a^	0.37 ± 0.01^A, a^	0.91 ± 0.05^A, a^	0.58 ± 0.09^A, a^
	6	5.72 ± 0.15^A, a^	0.38 ± 0.02^A, a^	0.94 ± 0.13^A, a^	0.59 ± 0.11^A, a^
	8	5.63 ± 0.24^A, a^	0.37 ± 0.03^A, a^	0.96 ± 0.11^A, a^	0.60 ± 0.09^A, a^
CS‐SW in burger	0	5.49 ± 0.17^A, a^	0.39 ± 0.03^A, a^	0.88 ± 0.09^A, a^	0.57 ± 0.07^A, a^
	2	5.55 ± 0.22^A, a^	0.38 ± 0.03^A, a^	0.91 ± 0.06^A, a^	0.56 ± 0.10^A, a^
	4	5.56 ± 0.14^A, a^	0.39 ± 0.04^A, a^	0.94 ± 0.13^A, a^	0.60 ± 0.08^A, a^
	6	5.63 ± 0.18^A, a^	0.36 ± 0.02^A, a^	0.94 ± 0.07^A, a^	0.59 ± 0.08^A, a^
	8	5.70 ± 0.25^A, a^	0.38 ± 0.01^A, a^	0.97 ± 0.09^A, a^	0.63 ± 0.15^A, a^

*Note*: Values represent mean (*n* = 3) ± *SD* Small and big different letters indicate significant difference among samples and storage period at 5% level of probability, respectively.

Abbreviations: CS‐SCF, *Capparis spinose* essential oil obtained by CO_2_ supercritical fluid; CS‐SW, *Capparis spinose* essential oil obtained by subcritical water; CS‐WD, *Capparis spinose* essential oil obtained by water distillation; SE, sodium erythorbate.

### 3.8 Microbiological assessment

**FIGURE 3:**
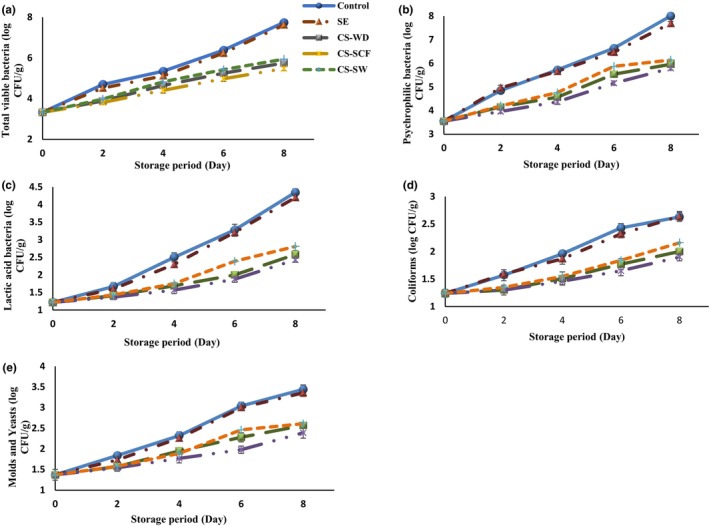
Changes in microorganism counts: (a) total viable bacteria, (b) psychrophilic bacteria, (c) lactic acid bacteria, (d) coliforms, and (e) molds and yeasts of fish burgers containing different essential oils during the storage period. CS‐SCF, *Capparis spinose* essential oil obtained by CO_2_ supercritical fluid; CS‐SW, *Capparis spinose* essential oil obtained by subcritical water; CS‐WD, *Capparis spinose* essential oil obtained by water distillation; SE, sodium erythorbate.

One of the most important issues for preserving fish burgers is the spoilage problem, which shortens the shelf life of these products (Eghbalian et al., 2021). Therefore, it is necessary to control the microbial growth and develop the quality and storage of the fishery products (Rico et al., 2020). In this research, the microbiological characteristics of fish burgers were studied by counting total viable bacteria (TVBC), psychrophilic bacteria, coliforms, LAB, molds, and yeasts during the 8 days of refrigerated storage (Figure 3). The acceptability limits were detected <7 log CFU/g, <7 log CFU/g, <400 log CFU/g, <4 log CFU/g, and <3 log CFU/g for TVBC, psychrophilic bacteria, coliforms, LAB, molds, and yeast counts, respectively (Boulares et al., 2018); in Figure 3, this acceptance limit is marked as red dotted lines.

The initial TVBC, psychrophilic bacteria, coliforms, LAB, molds, and yeast counts were 3.33, 3.55, 1.24, 1.22, and 1.37 Log CFU/g, respectively. The population of the mentioned microorganisms improved remarkably over time in all fish burgers (*p* < .05). Any preservative constituents were not added; therefore, the highest microorganism was observed in control; although, treated sample with SE had a good antioxidant activity and remarkable antimicrobial function. However, the addition of CSEOs to the fish burger formulation was able to significantly reduce microorganism growth due to bioactive constituents in CS (*p* < .05). Among the distinct forms of CS, the highest microbial feature was related to fish burgers with CS‐SCF, and no statistical difference was observed between other treated samples; however, treatments achieved by CS‐SW significantly have less antimicrobial activity than other forms of CS (*p* < .05). Overall, on the last day of refrigerated storage, the highest TVBC, psychrophilic bacteria, coliforms, LAB, molds, and yeasts were observed in control (7.74, 8.00, 2.63, 4.35, and 3.44 Log CFU/g), followed by burger including SE (7.62, 7.69, 2.64, 4.20, and 3.36 log CFU/g), respectively. The lowest microorganism was detected in the sample with CS‐SCF (5.48, 5.79, 1.90, 2.43, and 2.39 log CFU/g, respectively) on the 8th day. Nevertheless, there was no significant difference between the microbial loads of burgers containing SE and CSEOs. Antimicrobial constituents in natural preservatives (plant EOs) enter the lipids of the cell membrane and mitochondria, changing cell structure and increasing their permeability (Hematian et al., 2020; Olivas‐Méndez et al., 2022). It was demonstrated that ions and other materials in cells leak, and death occurs due to the release of vital molecules (Ji et al., 2021; Jooyandeh & Yadmellat, 2018). Previous research exhibited that antimicrobial traits of active substances depended on their molecular structure and concentration (Amiri et al., 2019; Nouri, 2022), and this feature of CS had been confirmed against molds and Gram‐positive and Gram‐negative bacteria (Hamatian et al., 2020). It was reported that the microbial load for salmon fish burgers was reduced owing to the addition of oregano and clove EOs (Jonušaite et al., 2021). A reduction in total microbial and psychrophilic bacteria was observed by adding *Zataria* and marjoram EOs to fish surimi compared with control (Pourmollaei et al., 2021). The presence of Lavendula and Moringa extracts in tilapia fish burgers could improve microbial shelf life of products for at least 7 days (Delfino et al., 2023).

### 3.9 Sensory evaluation results

Sensory characteristics are important parameters that have a noticeable impact of food product acceptance by consumers (Mooliani & Nouri, 2021; Shahbazi & Shavisi, 2018).

**FIGURE 4:**
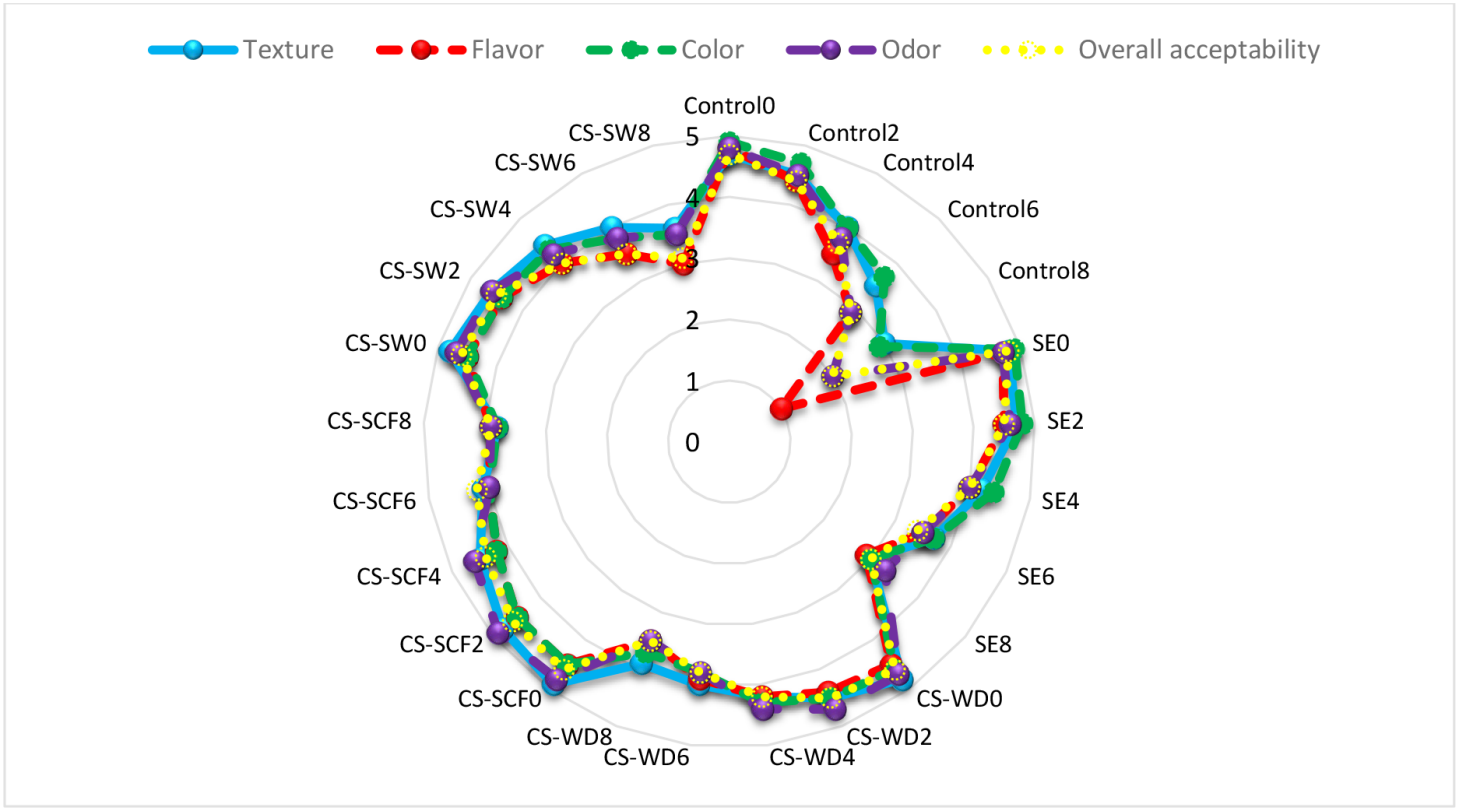
Comparison of sensory characteristics scores of fish burgers containing different essential oils during the storage period. Control_0_ to Control_8_: Control on 0, 2, 4, 6, and 8 days of storage/SE_0_ to SE_8_: Burger containing sodium erythorbate on 0, 2, 4, 6, and 8 days of storage/CS‐WD_0_ to CS‐WD_8_: Burger containing *Capparis spinose* essential oil obtained by water distillation on 0, 2, 4, 6, and 8 days of storage/CS‐SCF_0_ to CS‐SCF_8_: Burger containing *Capparis spinose* essential oil obtained by CO_2_ supercritical fluid on 0, 2, 4, 6, and 8 days of storage/CS‐SW_0_ to CS‐SW_8_: Burger containing *Capparis spinose* essential oil obtained by subcritical water on 0, 2, 4, 6, and 8 days of storage.

Overall, undesirable changes led to microbial growth, fat oxidation and decomposition of protein structure during storage and also sensory acceptance was reduced by consumers (Amiri et al., 2019). This was due to changes in color, taste, odor, and texture that were associated with a decrease in shelf life of the product (Aminzare et al., 2022). The results of sensory characteristics for fish burgers are depicted in Figure 4, and there was no significant difference in scores, which were 4.40–4.90 on the 1st and 2nd days. During refrigerated storage, this factor for fish burgers gradually decreased, but the reduction in control was higher than for burgers containing additives due to fat oxidation and microbial growth (*p* < .05). The control followed by the sample, including SE, had the lowest sensory scores, and the highest score was observed in burgers with CSEOs on the 8th day of storage. In general, CSEOs lowered fat oxidation, delayed microbial growth, and preserved the sensory characteristics of fish burgers over time. A significant influence was not found on this attribute using rosemary, thyme and clove EOs in fish patties on the 1st day; however, these additives were able to preserve sensory acceptance during period (Guran et al., 2015). The sensory maintenance of catfish burgers was also reported by adding *Zataria multiflora Boiss* EO (Çoban & Keleştemur, 2017), and sensory acceptance was observed for Nile tilapia fillets owing to use of water lily extracts (Dulal et al., 2023).

According to sensory attributes, rainbow trout fillets treated with *Mentha spicata* EO (0.4%) and zinc oxide nanoparticles (0.2 and also 0.4%) demonstrated the best score in terms of refrigerated shelf life (Shahbazi & Shavisi, 2018).

## 4 CONCLUSION

The results of the present research demonstrated that among three distinct methods of extraction, CS‐SCF with a significant difference had the highest amount of TPC (9.13 mg GAE/g), TFC (5.62 mg QE/g), and DPPH radical scavenging (73.24%) compared with others. CSEOs (0.2%) could considerably reduce pH, fat oxidation, and TBA for fish burgers compared with controls during 8 days. A significant influence was not distinguished on textural factors of control and treated burgers by adding CSEOs, which illustrated lower brightness with higher redness and yellowness compared with others. However, a remarkable impact was found between the antimicrobial function in control and burgers, including CSEOs, that total viable bacteria (<7 CFU/mL), psychrophilic bacteria (<7 CFU/mL), and molds and yeasts (<3 CFU/mL) did not exceed the acceptability limit in these treated samples until the end of storage. In addition, CSEOs were maintained sensory quality and acceptability of treated burgers towards to control. As a conclusion, the results of the present research depicted that samples including CS‐SCF could be successfully used to develop the oxidative and microbial shelf life as well as maintain the quality and safety of fishery products.

## AUTHOR CONTRIBUTIONS

Mohammad Shafaghi Rad: Conceptualization, Software, Validation, Formal assessment, evaluation, Resources, Information curation. Marjan Nouri: Methodology, Writing – original draft and review, Software editing.

## CONFLICT OF INTEREST STATEMENT

No conflicts of interest were declared by the authors.

## Data Availability

The information will be available from the corresponding authors.
